# Does real estate bubble affect corporate innovation? Evidence from China

**DOI:** 10.1371/journal.pone.0257106

**Published:** 2021-09-10

**Authors:** Chen Wang, Xiaowei Ma, Hyoungsuk Lee, Zhen Chu

**Affiliations:** 1 School of Economics, Fujian Normal University, Fuzhou, China; 2 East Asia Environment Research Center, Inha University, Michuhol-gu, Incheon, Korea; 3 School of Economics and Management, Tongji University, Shanghai, China; Istanbul Medeniyet University: Istanbul Medeniyet Universitesi, TURKEY

## Abstract

With the rapid increase of downward pressure on China’s economy, the stability of the property market, as an important part of the economic transformation process, also has a far-reaching impact on enterprises’ R&D investment. We select the data of Chinese large and medium-sized industrial enterprises from 1998 to 2015 as our research sample and propose a new combination measurement model based on closeness degree to measure the real estate bubble level in China accurately. The structural vector autoregressive (SVAR) theory is utilized to empirically test the dynamic relationship between the real estate bubble, corporate liquidity, and R&D investment. The results indicate that the real estate bubble level in China is increasing, and a certain risk of deviating from the safety interval in the future exists; The rapid expansion of the real estate bubble has a continuing negative impact on corporate R&D investment, that is, its "credit mitigation effect" is much smaller than the "capital relocation effect," and industrial enterprises will fall into the so-called "low-tech lock-in" state. In other words, to a certain extent, the development of this kind of real estate bubble will not be conducive to the transformation and upgradation of enterprises and long-term economic growth.

## Introduction

Since the reform and opening-up, China’s economy has grown rapidly at an average annual rate of nearly 10%, creating a "growth miracle" that has attracted global attention. In recent years, China’s economy has entered a state of "new normal," featuring a significantly slowing economic growth [1, 2]. The economic growth rate in 2018 was only 6.6%. However, this extensive growth has also brought a series of environmental problems, which have seriously harmed the health of residents and the efficiency of economic operations. According to the China Meteorological Administration, many cities in central and eastern China have long been plagued by smog, with an average of 35.9 days in 2013—the most since 1961—and some cities having more than 100 days of smog. As a result, more than one-fifth of the Chinese population’s medical expenditure is devoted to preventing and treating diseases caused by environmental pollution. The economic loss caused by environmental problems every year amounts to 10% of the GDP (data obtained from a 2011 report by the Chinese Academy of Environmental Sciences and calculations by the World Bank). In the context of increasing constraints on environmental resources and the gradual loss of demographic dividends, the downward pressure on China’s economy is mounting. The factor-driven pattern of growth that it has depended on in the past will become unsustainable. In most developing countries, government-led political regulation of energy efficiency and urban climate adaptation is likely to be more effective, at least initially. Still, it cannot be sustained without strong private sector support. The initial stages of ecological civilization saw many policy failures. As a form of soft regulation, the public environmental concern makes corporate management more focused on environmental responsibility [3]. The new investments or innovate-products, in turn, support environmental management [4]. Therefore, enterprises’ green growth model development is important in coordinating economic development and environmental protection solutions. This study focuses on the technological innovation challenges faced by enterprises under the Paris system in 2020.

**Fig 1:**
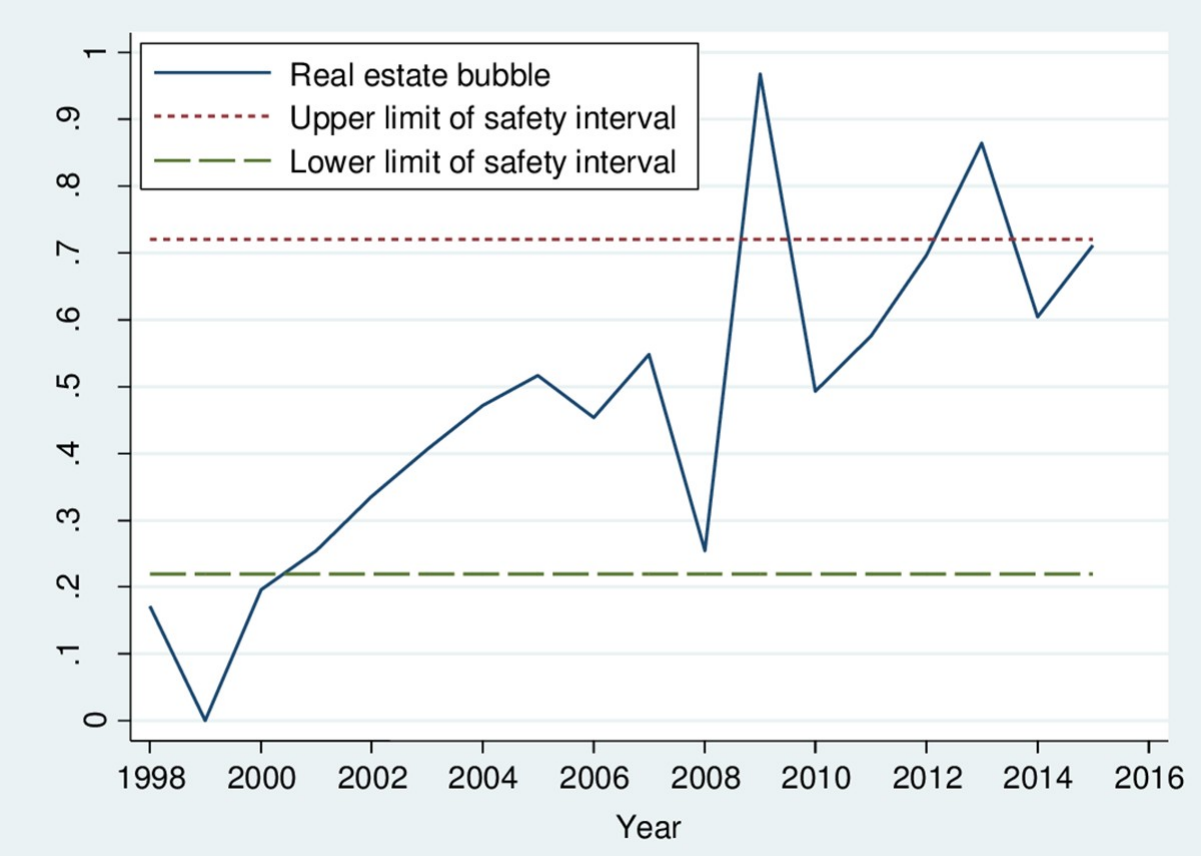
Trend chart of the real estate bubble from 1998 to 2015 in China. Note: The imposed safety interval of the real estate bubble is based on the prevailing 3σ method.

The endogenous growth theory shows that R&D investment and innovation capabilities help improve economic efficiency and increase China’s potential economic growth rate [5]. Therefore, during the economic transition period, China needs to pay more attention to the R&D investment by enterprises. From the traditional R&D perspective, the effective demand scale promoted by rapid economic growth will help increase R&D investment [6, 7]. According to the database of Chinese industrial enterprises, although the overall R&D intensity in China shows an increasing annual growth trend, there is still a considerable gap compared with that in developed countries [8, 9]. Specifically, the innovation investment of industrial enterprises is even lower than the optimal R&D scale. China’s industrial enterprises have entered into the so-called "low-tech lock-in" state [10]; that is, the insufficient R&D investment of enterprises does not match the rapid economic development in China (see Fig 1). Similarly, since the Chinese government abolished the housing distribution system in 1998, the real estate industry began to develop rapidly and has shown a tendency to bubble. Specifically, the real estate investment demonstrates a relatively large scale and fast growth rate; housing prices have seen a sharp rise; compared with the average profit rate of industrial enterprises of only 7.4%, real estate enterprises have an average profit margin as high as 28.7%. Driven by individual rational decision-making, the formation and expansion of the real estate bubble inevitably attract much social capital. First, entity companies have the motivation to enter the real estate industry for cross-industry arbitrage, which is bound to have a significant crowding effect on corporate investment, especially R&D investment, under conditions of financial constraints. Second, the commercial banking system provides limited funds to the real estate sector with low risk and high yield. In the Chinese financial system, dominated by banks, the increase of the external financing costs of physical enterprises has caused a significant crowding effect on the long-term investment required for innovation. Finally, the bulk of household savings is spent on purchasing real estate industries where innovation activities are relatively low. Therefore, the demand for high-tech products with more innovation activities declines, leading to the failure of the "innovation caused by domestic demand" function and reducing enterprise incentive for R&D and innovation.

Miao and Wang [11] constructed a two-sector endogenous economic growth model with and without technology spillovers, combining observations on the Chinese economy (corresponding to industry and real estate in this study). The model assumes that all companies have financing constraints and asset bubbles only exist in sectors without technology spillovers (i.e., the real estate industry). The theoretical derivation indicates that speculative bubbles cause capital redistribution among different production departments (that is, attracted by the real estate bubble, non-real estate companies will invest in the real estate industry, thereby inhibiting the innovation investment in the main business). The expansion of the real estate bubble on the R&D investment for industrial enterprises is mainly divided into positive and negative. The first mechanism is the credit mitigation effect, an innovative growth mechanism ignored by many studies. The credit mitigation effect means that companies can increase the value of mortgage resources such as enterprise-owned houses and land through high housing prices in areas with high levels of real estate bubbles. It has certain advantages in external financing, which can alleviate the financing constraints and be conducive to investment scale expansion and R&D investment increase of enterprises [12, 13]. The second mechanism is the resource relocation effect. It is well known that capital is profit-driven. The high return on investment driven by the real estate bubble will induce industrial enterprises to carry out cross-industry arbitrage, thereby increasing this kind of capital investment with low risk and quick results in the short term. Especially in the face of financing constraints, companies are bound to reduce R&D investment accordingly to reduce investment losses [8, 10, 14]. Therefore, whether the real estate bubble can finally restrain the innovation investment of enterprises depends on the dominant effect. It is hence necessary to further investigate the deep-seated incentives that influence the cross-industry arbitrage motivation of enterprises. The period of rapid economic growth in China is also a rapid expansion of the real estate bubble. Consequently, it is of utmost significance to correctly understand the dynamic mechanisms of the property bubble, corporate financing constraints, and enterprise R&D investment, and coordinate the relationship among these three, which provides a new perspective for the reform of economic development in the stage of the new normal [11, 15].

Regarding the measurement of the real estate bubble, there are currently two main research ideas prevalent at home and abroad: the direct measurement approach and the indirect measurement approach. As the name implies, the direct measurement method is a process of theoretically evaluating real estate based on related models, comparing it with the actual price, and finally measuring the degree of the bubble. The indirect measurement method is categorized into two types. One is to select single or multiple indicators that can reflect the real estate bubble and simply judge whether there is a real estate bubble based on the gap between the actual indicator value and the critical value of the indicator. Base on relative rental price index of three major cities of Turkey namely, Istanbul, Izmir, and Ankara during the period from 1994 to 2010, Bilgin et al. [16] find there is a nonlinear rental price convergence in Turkish property market using a new non-linear panel ADF test. The other is based on a multi-dimensional indicator system reflecting the real estate bubble, and then matching the actual value of each indicator with its corresponding authoritative measurement segment to obtain the respective measurement value. Finally, according to the given authoritative index weights, the sum of the weighted value of each measurement is given. Then, the operation status of the real estate bubble can be measured [17–20]. Regarding the research on interaction mechanisms among the real estate bubble, financing constraints, and corporate R&D investment, based on microdata, Gan [21] suggested that the real estate bubble can increase the value of corporate collateral to a certain extent, enhance its external financing capabilities and ease financing constraints, and consequently drive the R&D investment of industrial enterprises (i.e., the "credit mitigation effect" of the real estate bubble) [22–24]. By establishing a two-sector endogenous economic growth model, Shi et al. [25] found that the asset sector with bubbles would attract the entity production sector with financing constraints, thereby reducing R&D investment driven by cross-industry arbitrage motivation (i.e., the "capital reallocation effect" of the real estate bubble). If this type of bubble sector does not have technology spillover effects (such as the real estate industry), this "capital reallocation effect" will negatively impact economic development. Recently, similar to the research on the mechanism of the real estate bubble, Rong et al. [26] empirically tested the mechanism of high housing prices on R&D investment of industrial enterprises and new product output based on a panel data model. The research showed that the rapid rise in housing prices would inhibit the R&D investment of industrial enterprises. Based on the characteristics of excess profit margins in the Chinese real estate market, Wang et al. [27] empirically analyzed the cross-industry arbitrage behavior of entity companies and the corresponding impact on innovative research and development.

Based on our review of related literature, there are still some shortcomings in most of the current studies. Regarding the measurement of the real estate bubble, the unavailability of data increases the difficulty of direct measurement method in practice. However, the indirect measurement method division of indicator measurement segments and indicator weights rely mainly on foreign standards or scores based on relevant experts. Accordingly, the measurement results are more susceptible to subjective bias. A few studies explore the interaction mechanism between the real estate bubble, corporate financing constraints, and corporate R&D investment, but with more theoretical explanations on the relationship among these three, and with little empirical analysis. Furthermore, most of the literature on empirical tests only bases itself on the perspective of rising housing prices, simply expounding the relationship between rising housing prices and corporate innovation, instead of incorporating the housing bubble, which can more comprehensively describe the operation of the real estate market with other factors as a unified framework for analysis. Based on the preceding considerations, this study attempts to construct a combined measurement model based on the vector angle cosine (multivariate statistical method), providing a valuable reference for the accurate measurement of the real estate bubble. This study employs the structural vector autoregression (SVAR) theory to dynamically test the negative economic effects of the real estate bubble development and emphasizes the negative impact of the real estate bubble expansion on the transformation and upgrading of enterprises and long-term economic growth.

The rest of this paper proceeds as follows. Section 2 introduces a new hybrid, multiple attribute decision-making (MADM) method and the structural vector autoregressive (SVAR) model. Section 3 presents the variables’ design and a description of the data and then offers an empirical analysis of the data. Finally, we provide concluding remarks in Section 4.

## Methodology

### General Weighted Average (GWA) operator combination measurement model based on closeness degree

In evaluating complex economic systems, a single evaluation method is often too one-sided. In contrast, a combined evaluation model is considered a workable solution to address this issue. Therefore, this study proposes a new combination evaluation model based on closeness degree—GWA operator combination measurement model based on closeness, to improve the scientific rationality of the evaluation [28–31].

Suppose we apply *m* individual measurement methods to *n* measurement units. After standardization, we obtain the single measurement result matrix *A* = {*x*_*ij*_}_*n*×*m*_, where *x*_*ij*_ is the measured value obtained for the *i*-th measurement unit by the *j*-th measurement approach. Different from the prediction problem, the "true value" of the measured object is unobservable, causing great inconvenience in addressing the optimal combination issues. However, if we assume that "true value" exists in the individual measurement results with a certain probability, then the optimization scheme can be converted into a problem of minimizing the sum of the "distance" between the "combined value" and each individual measurement value. Furthermore, for such a "distance" measurement between data sequences, the correlation optimization criterion based on closeness degree is more advantageous. This study attempts to construct a GWA operator combination measurement model based on closeness degree to integrate the individual measurement results effectively. The specific structure is given as follows.

$$\begin{array}{l} \max S(W)=\sum_{j=1}^{m}\tau_{j}(W)=\sum_{j=1}^{m}\left(1-\frac{\sum_{i=1}^{n}{\left(x_{i}(W)-x_{ij}\right)}^{2}}{\sum_{i=1}^{n}x_{i}{\left(W\right)}^{2}+\sum_{i=1}^{n}x_{ij}{}^{2}}\right)\\ s.t.\left\{ \begin{array}{l} x_{i}(W)={\left(\sum_{j=1}^{m}w_{j}x_{ij}^{\lambda }\right)}^{1/\lambda }i=1,2,\cdots ,n\\ \sum_{j=1}^{m}w_{j}=1\;w_{j}\geq 0,j=1,2,\cdots ,m \end{array}\right. \end{array}$$ (1)

where *η*_*j*_(*W*) indicates the closeness degree for the combined measurement approach, *W* = [*w*_1_,*w*_2_,⋯,*w*_*m*_]^*T*^ is the weight vector for each individual measurement method and *x*_*i*_(*W*) represents the general weighted average (GWA) combined measurement value of the *i*-th measurement unit.

To evaluate the effectiveness of the proposed combined evaluation model based on closeness degree, we construct an error evaluation index system with the square sum of error (SSE), mean square error (MSE), mean absolute error (MAE), mean absolute percentage error (MAPE), and mean square percentage error (MSPE) for validity demonstration. The true value of the evaluation problem is more difficult to determine than the prediction problem. According to the central limit theorem, we take the average sequence of each method as the benchmark evaluation value of the *i*-th evaluation unit in the absence of a better alternative. Notably, to ensure the rationality and effectiveness of the combined evaluation results, the employment of the combined evaluation method based on several single evaluation methods is required to meet two important conditions: on the one hand, several single evaluation results are consistent and can be mutually verified; on the other hand, the obtained combined evaluation is supposed to be closely related to the original evaluation result. Therefore, it is necessary to use the Kendall consistency coefficient test to perform pre- and post-test in the combination evaluation.

### Structure vector autoregressive (SVAR) model

Sims first proposed the vector autoregressive (VAR) model in 1980 to solve weak predictive ability and difficulty in model identification. However, some problems have emerged in this simplified VAR model. For example, its impulse response function is not unique (depending on the order of variables). However, the current impact (economic structure) between response variables cannot be revealed. Recently, a Quantile Structural Vector Autoregressive (QSVAR) model has been applied to examine the effect of monetary policy shocks on growth rate of real house price, and to assess the real effects of uncertainty shocks in expansions and recessions in the United States [32, 33]. Therefore, it is necessary to re-incorporate the structure into the VAR model to form the so-called "structural VAR" technique [34–36].

Generally, the expression of the simplified VAR of order p can be formulated as follows:

$$Y_{t}=\Gamma_{1}Y_{t-1}+\Gamma_{2}Y_{t-2}+\cdots +\Gamma_{p}Y_{t-p}+u_{t}$$ (2)

where *Y*_*t*_ is a *M*×1 vector and *u*_*t*_ is the simplified disturbance allowing for the contemporaneous correlation. Then, by multiplying both sides of Eq (2) by some non-degenerate matrix *A*, we obtain:

$$AY_{t}=A\Gamma_{1}Y_{t-1}+A\Gamma_{2}Y_{t-2}+\cdots +A\Gamma_{p}Y_{t-p}+Au_{t}$$ (3)

After transposition, we obtain:

$$A(I-\Gamma_{1}L-\Gamma_{2}L^{2}-\cdots -\Gamma_{p}L^{p})Y_{t}=Au_{t}=B\varepsilon_{t}$$ (4)

where *ε*_*t*_ is the structural disturbance of SVAR with no contemporaneous correlation and covariance matrix normalized to the identity matrix *I*_*M*_. Eq (4) is called the "AB model" of SVAR. It is necessary to impose (2*M*^2^−*M*(*M*+1)/2) constraints on the elements in matrices *A* and *B* to identify the AB model accurately. Therefore, the technique in common use is the "Cholsky constraint." We set *B* as a diagonal matrix and *A* as a lower triangular matrix with main diagonal elements all equal 1. As the estimation result depends on the order of the variables, it should be combined with relevant theoretical elaborations or consider the selected specific variable order comprehensively through the sensitivity analysis in the specific operation process.

## Empirical analysis

### Data

By analyzing the indicators adopted by domestic and foreign scholars regarding the early warning of the real estate bubble and combining with the current situation in China, we screen out an indicator system that can effectively forewarn the real estate bubble composite index (denoted as PM; we build a PM calculating the values of a comprehensive index by using a combination evaluation method). To build a comprehensive evaluation index framework of real estate bubble, researchers have conducted a holistic examination through reviews of the literature. The indicators have usually been selected and then categorised into three groups: supply side risk, demand side risk, and financial risk. Table 1 presents the established evaluation index system for the real estate bubble in China. Specifically, we use the proportion of industrial enterprise R&D expenditures to total assets (denoted as QC) to measure the industrial enterprise R&D investment. Additionally, we use the expression "(industrial enterprise current assets-industrial enterprise current liabilities)/total assets" (denoted as RY) to present the status of industrial enterprise liquidity (financing constraints).

**Table 1:** Evaluation index system for the real estate bubble in China.

Dimension	Indicator	Obs	Mean	Std.	Min	Max
Supply side risk	SR1. The proportion of real estate development investment in GDP (%)	18	9.48	3.54	4.26	14.94
	SR2. The ratio of construction area to completed area of commercial housing	18	4.22	1.59	2.62	7.35
	SR3. The proportion of real estate development investment in the total investment in fixed assets of the whole society (%)	18	17.39	1.89	12.72	19.84
Demand side risk	DR1. House price to income ratio	18	0.45	0.10	0.31	0.63
	DR1. The ratio of real estate price growth rate to GDP growth rate	18	0.59	0.59	-0.09	2.54
Financial risk	FR1. Growth rate of domestic loans of real estate development companies (%)	18	19.70	15.25	-4.84	49.42

Note: This table presents the evaluation index system for the real estate bubble in China. To mitigate the influence of outliers, all of the ratio variables are winsorised at the 1% level in a two-tailed test.

Referring to the China Statistical Yearbook, China Real Estate Statistical Yearbook, and China Industrial Enterprise Economic Statistical Yearbook, we obtain the statistical data corresponding to various indicators from 1998 to 2015 in China and construct an original evaluation data matrix of the real estate bubble composite index $\hat{X}={\left({\hat{x}}_{ij}\right)}_{18\times 6}$, the original time series of the mobility, and the R&D investment of large and medium-sized industrial enterprises. Furthermore, to eliminate the influence of severe fluctuations and heteroscedasticity in the time series during empirical modeling, we use the logarithm of the three variables and obtain ln*PM*_*t*_, ln*RY*_*t*_, and ln*QC*_*t*_ respectively.

### Combination measurement of real estate bubbles

Step 1: Construct a dimensionless original sample matrix ${\left\{x_{ik}^{\prime }\right\}}_{n\times p}$, where $x_{ik}^{\prime }$ indicates the *k*-th index value corresponding to *i*-th evaluation unit. We select a reasonable evaluation index system considering the special circumstances of the real estate bubble measurement in China and conduct the dimensionless treatment for the original sample matrix based upon the index attributes, thereby effectively eliminating the incommensurability caused by different dimensions units. Then we calculate the normalized single measure matrix {*x*_*ij*_}_*n*×*m*_, where *x*_*ij*_ represents the *j*-th single measurement value corresponding to the *i*-th measurement unit. Each indicator is the main influencing factor that affects the degree of the bubble in real estate, but not every indicator plays an equal role in the corresponding measurement. Therefore, we select the entropy and factor analysis methods based on objective and quantitative perspectives to effectively integrate the measurement information of each indicator and the analytic hierarchy process based on subjective and qualitative perspectives to measure the real estate bubble separately. Furthermore, the evaluation matrix is normalized according to the single evaluation method, which effectively eliminates the impact on the combined effect caused by the large gap between the results of various approaches.

Step 2: Calculate the combined measure value *x*_*i*_(*W*) based on the vector angle cosine according to formula (1). It is well known that the entropy method that relies on sample data is often affected by the randomness of the data. The factor analysis approach inevitably loses part of the original information in comprehensively concentrating the indexes. Moreover, the section of expert scoring often shows a certain degree of subjectivity and arbitrariness for the analytic hierarchy. Therefore, to make up for the one-sidedness of the individual measurement models and synthesize various aspects of information more effectively, it is important to integrate these three evaluation methods. First, we use Kendall’s consistency coefficient to test the compatibility of the three single measurement methods mentioned in Step 2. The Kendall consistency coefficient test results indicate that these three particular evaluation methods are consistent at the significance level *α* = 0.01. That is, the obtained results of the three measurement methods with different evaluation angles are closely related. The validity of each of the three methods has been verified, providing the premise for the following combination measurement. Then, based on the generalized weighted combination evaluation model of the vector angle cosine, we combine the above three compatible single measurements and obtain the corresponding weight vector [w_1_, w_2_, w_3_]^T^ = [0.318,0.287,0.395]^T^. The combined measurement values of the real estate bubble are then calculated and illustrated in Fig 1.

Finally, we use the Kendall consistency coefficient to test the degree of closeness between the combined evaluation method and various single, compatible evaluation methods. The Kendall consistency coefficient test results indicate that the three single evaluation methods and the combined evaluation methods are consistent at the significance level *α* = 0.01. Specifically, the real estate bubble degree from 1998 to 2015 in China is highly correlated under these four evaluation techniques. Therefore, the post-test of the combined measurement method has been verified. Meanwhile, through the calculation and comparison among the effectiveness evaluation values under each measurement method, it is found that the error-index values of the combined measurement model based on the vector angle cosine are much smaller than that of the other single measurement approaches, as depicted in Table 2. This further corroborates that the combined measurement result based on the vector angle cosine is better than the other single measurement results. Therefore, it can portray the overall situation of the real estate bubble in China well. So far, based on measuring the composite index of the real estate bubble in China accurately, we have a clearer picture of the operating status of the real estate. By reviewing its evolution, we find that the period of rapid economic growth in China also emerges as the period of rapid expansion of the real estate bubble [20]. However, the bubble theory shows that the continuous expansion of the real estate bubble is not conducive to developing certain aspects of the real economy. We use the SVAR model in the following section to conduct the empirical research on the dynamic mechanism of the real estate bubble, corporate liquidity, and industrial enterprise R&D investment, and therefore explain industrial enterprises’ fall into the "low-tech lock-in" status from the perspective of the real estate bubble.

**Table 2:** Comparison of the effectiveness of measurement models.

Effectiveness evaluation index	SSE	MAE	MSE	MAPE	MSPE
Entropy method	0.097	0.017	0.058	0.124	0.035
Factor Analysis	0.244	0.027	0.094	0.222	0.073
Analytic Hierarchy Process	0.461	0.038	0.119	0.248	0.078
Combination evaluation method	0.004	0.004	0.012	0.025	0.008

Note: This table presents an error evaluation index system with square sum of error (SSE), mean square error (MSE), mean absolute error (MAE), mean absolute percentage error (MAPE), and mean square percentage error (MSPE).

### Dynamic analysis of SVAR model

#### Correlation test of variables

This s determines the optimal lag order based on the AIC criterion to construct a regression model with intercept or trend terms to avoid the pseudo-regression phenomenon that may exist in the time series regression. It then employs the ADF-fisher technique to test the stability of the variables. Table 3 presents the test results. It is clear that ln*PM*_*t*_, ln*RY*_*t*_, and ln*QC*_*t*_ are all 1st-order single integer sequences at the 5% confidence level.

**Table 3:** Unit root test results of each variable.

Variable	(c,t,p)	ADF statistics	1% critical value	5% critical value	10% critical value	Test result
ln PM	(1,1,2)	-2.42	-4.73	-3.76	-3.32	Non-stationary
ln RY	(1,1,1)	-3.00	-4,67	-3.73	-3.31	Non-stationary
ln QC	(1,1,5)	-1.63	-4.99	-3.88	-3.39	Non-stationary
△ln PM	(1,0,2)	-3.50	-4.00	-3.10	-2.69	Stationary
△ln RY	(1,0,1)	-3.90	-3.96	-3.08	-2.68	Stationary
△ln QC	(0,0,5)	-3.00	-2.79	-1.98	-1.60	Stationary

Note: c, t, and p represent the constant term, the time trend term, and the adopted lag order, respectively; the AIC criterion determines the value of p; μ represents the first difference of the variable sequence.

The SVAR model can be established directly for the corresponding analysis in stationary time series. But for non-stationary time series of the same order and single integral, it is necessary to set reasonable assumptions on the Johansen cointegration test method combined with the sensitivity of the trend hypothesis test results, and thus the cointegration relationship between variables can be clarified. Table 4 displays the obtained test results.

**Table 4:** Johansen cointegration test results for each given variable.

Maximum number of cointegration vectors	Trace statistics	5% critical value	Probability value	Maximum eigenvalue statistics	5% critical value	Probability value
0*	55.23300	29.79707	≤0.001	38.87184	21.13162	0.0001
1*	16.36116	15.49471	0.0370	11.86703	14.26460	0.1157
2*	4.494130	3.841466	0.0340	4.494130	3.841466	0.0340

Note: n* represents rejecting the null hypothesis at the 5% confidence level. There are at most n cointegration vectors in the three-variable system.

It is suggested that at the 5% significance level, the results of the race statistics test and the maximum eigenvalue test indicate that the system has two cointegration vectors. Therefore, the stable equilibrium relationship in the model has been proved. Furthermore, according to Sims [37], the SVAR model established based on their level values can be identified for several variables with the cointegration relationship. The corresponding least square estimation is consistent. Therefore, we also consider the model establishment based on level values to explore the short-term interactions of the endogenous variables in the system.

#### Model-identification and estimation

Before specifying the SVAR model, the optimal lag order has to be determined. This study employs five evaluation criteria, LR, FPE, AIC, SC, and HQ, to select the lag period in the SVAR model. The test results are given in Table 5.

**Table 5:** Selection of the optimal lag order.

Lag order	LR	FPE	AIC	SC	HQ
0	NA	6.64e-08	-8.014653	-7.873043	-8.016162
1	65.86274*	5.75e-10	-12.80218	-12.23574	-12.80821
2	9.123519	7.42e-10	-12.74262	-11.75135	-12.75317
3	12.79294	3.45e-10*	-14.10120*	-12.68510*	-14.11629*

Note

* indicates the selected lag order in each column of evaluation criteria.

Table 5 shows that the optimal lag order varies greatly under different criteria. The first lag is selected according to the LR criterion, while the third lag is selected based upon the AIC and FPE criteria. However, as proved in Luetkepohl [38], the AIC and FPE criteria tend to overestimate the lag order of the model. Therefore, by giving a careful consideration, this study attempts to construct a ternary SVAR (2) model for the real estate bubble, corporate liquidity, and corporate R&D investment. The specific structure is presented in formula (5).

$$A(I-\Gamma_{1}L-\Gamma_{2}L^{2})Y_{t}=Au_{t}=B\varepsilon_{t}$$ (5)

where *Y*_*t*_ = [ln*PM*_*t*_ ln*RY*_*t*_ ln*QC*_*t*_]^*T*^; *ε*_1*t*_, *ε*_2*t*_, and *ε*_3*t*_ represent the structural impacts (i.e., the structural residuals) on the real estate bubble, corporate liquidity, and R&D investment separately; *ε*_*t*_ is the white noise vector with covariance the identity matrix, that is, *ε*_*t*_~*VMN*(0,*I*_*n*_).

For accurate identification of the matrix *A* and matrix B in (5), this study utilizes the "Cholsky" approach to restrict *A* and *B* based upon synthesizing relevant economic assumptions and sensitivity analysis results. Specifically, considering the actual situation of the economic operation in China, the following three assumptions have been made on matrix *A*: (1) corporate liquidity will affect the current corporate R&D investment but will not exert an influence on the real estate bubble, that is, *a*_12_ = 0; (2) the real estate bubble will affect the corporate liquidity during the same period, but will not depend on the concurrent corporate R&D investment, that is, *a*_13_ = 0; (3) the concurrent corporate R&D investment may depend on the real estate bubble, but will have no significant impact on the corporate liquidity, that is, *a*_23_ = 0. Then, we apply the Full Information Maximum Likelihood Method (FIML) to estimate all the unknown parameters of the SVAR model and obtain the estimated results of the linear combination of *A*, *u*_*t*_, and *ε*_*t*_ as follows (the estimated residual of the VAR model is denoted as *u*_*t*_).


$$\left[\begin{array}{ccc} 1 & 0 & 0\\ -0.03 & 1 & 0\\ 0.11 & -0.53 & 1 \end{array}][\begin{array}{c} u_{1t}\\ u_{2t}\\ u_{3t} \end{array}]=[\begin{array}{c} \varepsilon_{1t}\\ \varepsilon_{2t}\\ \varepsilon_{3t} \end{array}\right]$$ (6)

**Fig 2:**
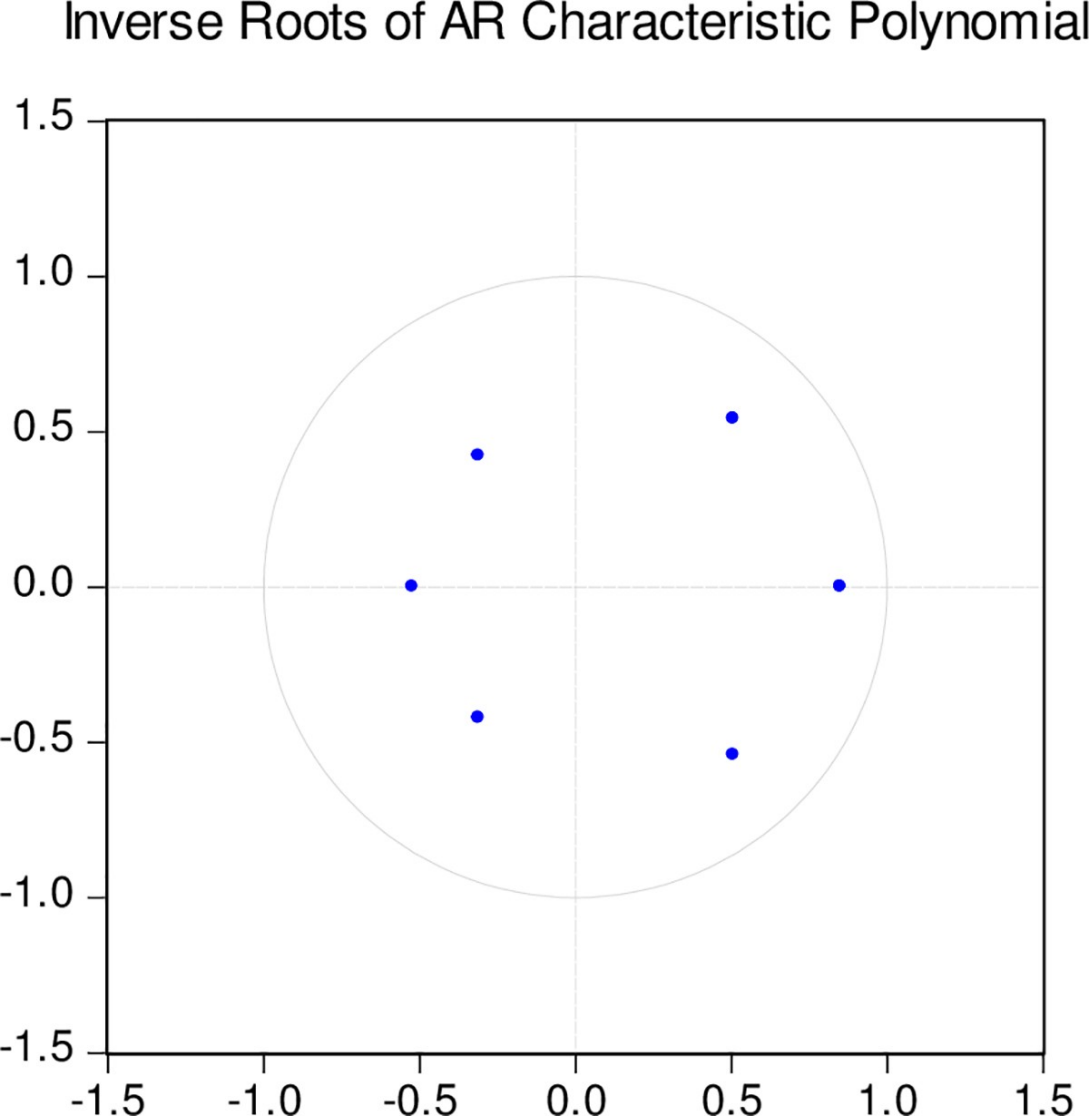
Discriminant diagram of SVAR system stability.

In the stationarity test (as depicted in Fig 2), all eigenvalues of the given SVAR (2) model are within the unit circle. Therefore, the system is considered stationary. Although some variables are not particularly significant, the primary focus of the SVAR (2) model is on the impulse response function and variance analysis. Therefore, the identified SVAR model can be utilized for further analysis of the subsequent related work.

#### Impulse response and variance decomposition

**Fig 3:**
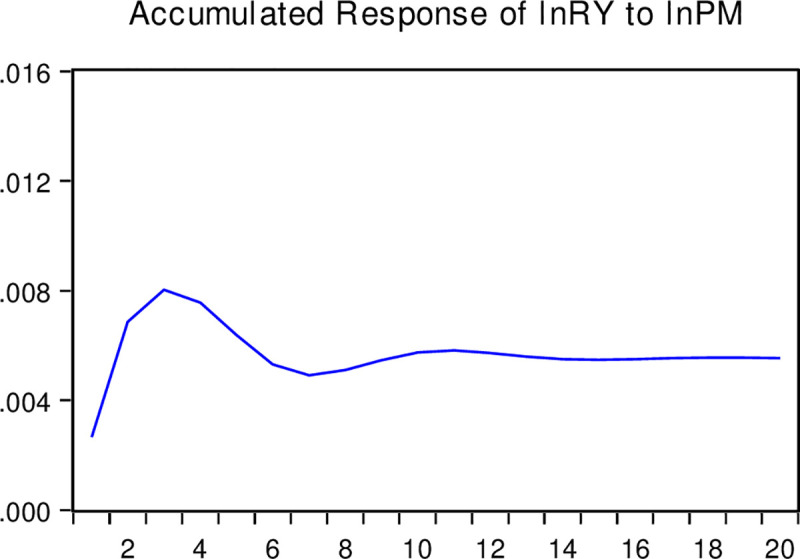
Accumulated response of lnRY caused by the structural impact of lnPM. Note: This figure presents the corresponding trajectories of the accumulated response respectively. The horizontal axis represents the time interval (years) after the impact, and the vertical axis indicates the degree of response (percentage) of each variable to the impact.

**Fig 4:**
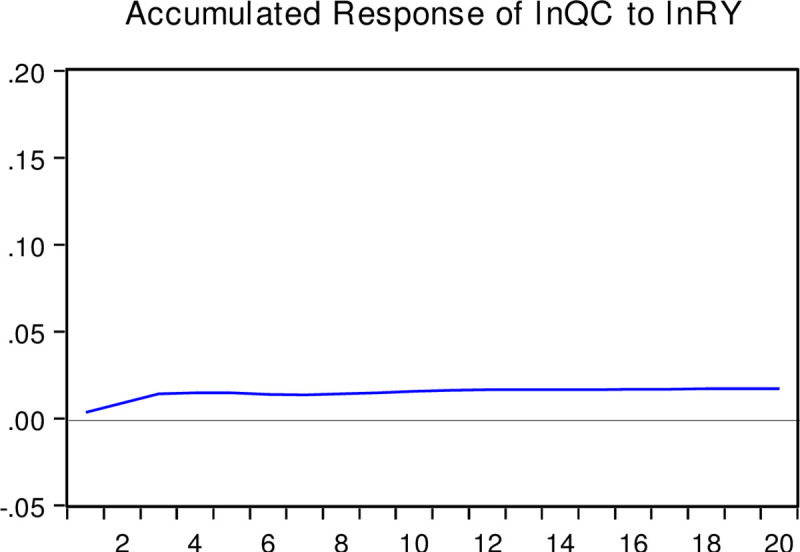
Accumulated response of lnQC caused by the structural impact of lnRY. Note: This figure presents the corresponding trajectories of the accumulated response respectively. The horizontal axis represents the time interval (years) after the impact, and the vertical axis indicates the degree of response (percentage) of each variable to the impact.

**Fig 5:**
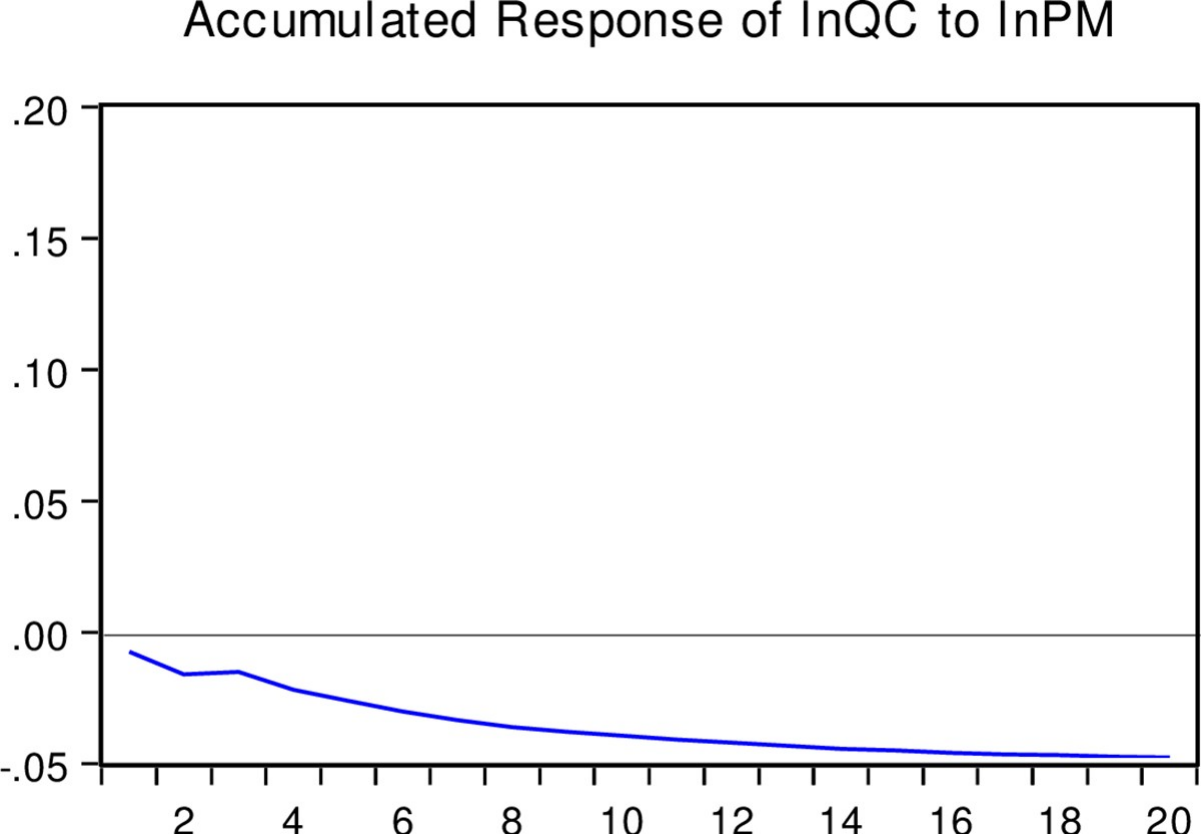
Accumulated response of lnQC caused by the structural impact of lnPM. Note: This figure presents the corresponding trajectories of the accumulated response respectively. The horizontal axis represents the time interval (years) after the impact, and the vertical axis indicates the degree of response (percentage) of each variable to the impact.

The impulse response function depicts changes in other endogenous variables caused by the impact of one endogenous variable through the dynamic structure of the SVAR model. In contrast, the variance decomposition technique usually relies on variance to measure the contribution of different structural impacts on the changes in endogenous variables. This study selects 20 years as the length of the lag period. We obtain the interactions among the real estate bubble, corporate liquidity, and corporate R&D investment through specific calculations. The response trajectory shows that the real estate bubble has a certain regular transmission mechanism for the R&D investment of enterprises. Figs 3–5 present the corresponding trajectories of the accumulated response, respectively. The horizontal axis represents the time interval (years) after the impact, and the vertical axis indicates the degree of response (percentage) of each variable to the impact.

- The responses of corporate liquidity to the real estate bubble: As interpreted from the accumulated impulse response function in Fig 3, the response of corporate liquidity to the accumulated impact of the real estate bubble is positive. Specifically, it rose rapidly in the first three years and reached a positive maximum of 0.008%. However, around the fourth year and after that, the accumulated impact response decreased slightly and then stabilized. This indicates that the positive impact of the real estate bubble is not very significant in promoting corporate liquidity, and this significant promotion is not sustainable.
- The response of corporate R&D investment to corporate liquidity: From Fig 4, it can be observed that the accumulated impact of corporate R&D investment on corporate liquidity is also positive. After the rapid rise in the previous three years and reaching the maximum positive value of 0.015%, the accumulated impact response stabilized. This shows that the influence of corporate liquidity impacts has a strong short-term positive effect but will not affect the R&D behavior of enterprises in the long run.
- The response of corporate R&D investment to the real estate bubble: From the accumulated impulse response function presented in Fig 5, it can be seen that the accumulated impact of corporate R&D investment on the real estate bubble is negative. After an accelerated decline in about the first 10 periods, the accumulated impact response gradually converged to around the negative 0.05%. Accordingly, the impact of the real estate bubble has a strong and sustained negative influence on corporate R&D investment.

We further compare the relative contribution rate of the real estate bubble and corporate liquidity to the R&D investment of enterprises using the variance decomposition technology—Table 6 displays the obtained variance decomposition results.

**Table 6:** The result of variance decomposition of corporate R&D investment.

Lag period	Impact of real estate	Impact of corporate liquidity	Impact of corporate R&D investment
1	4.558716	0.905939	94.53535
2	8.382063	2.520213	89.09772
3	6.732929	3.440566	89.82651
4	8.354263	3.121461	88.52428
5	8.363875	2.871077	88.76505
10	8.822999	2.590820	88.58618
20	8.849999	2.530875	88.61913

Note: The values in columns 2–4 represent the contribution rate (%) of one unit of exogenous impact to the influence of corporate R&D investment.

Table 6 shows that the level of corporate R&D investment has been affected by up to 88% of its impact in each period. Consequently, the contribution rate of its changes has far surpassed other impacts. This is probably due to the existence of many uncertain macro- and micro-factors that affect the level of corporate R&D investments. The contribution rate of changes in the real estate bubble reached 8.38% in the second period, that is, 8.38% of the prediction variance of changes in corporate R&D investment can be explained by changes in the real estate bubble. From the second period, the contribution rate of each period still showed an upward trend, with a slowing growth rate after the 10th period. However, the contribution rate of changes in corporate liquidity was only 3.44% in the third period, indicating that only 3.44% of the prediction variance of corporate R&D investment levels could be explained, after which it maintained a steady downward trend. Accordingly, the long-term impact of the real estate bubble on the corporate R&D investment has exceeded corporate liquidity.

Generally, the exogenous impact of the real estate bubble on the R&D investment of enterprises operates in the following two ways. First, the real estate bubble indirectly promotes the increase of corporate R&D investment by improving financing constraints of enterprises, the so-called "credit mitigation effect [21, 22]." However, under certain financing constraints conditions, the rate of return on property investment raised by the real estate bubble also causes industries to transfer part of their investment to the real estate industry, thereby weakening their R&D investment, the so-called “resource reallocation effect [27]." Based on the results of the impulse response and variance decomposition analysis presented above, it can be seen that the exogenous impact of the real estate bubble in China has an overall strong and long-term negative effect on the R&D investment of enterprises. However, it only exhibits a weak transient positiveness due to the "credit mitigation effect." Therefore, it can be easily validated that the "resource reallocation effect" of the real estate bubble in China is bound to show a strong negative effect in the long term [25, 26]. That is, with other factors remaining the same, the "credit mitigation effect" of the real estate bubble in China will be offset by part of the "resource reallocation effect," emerging as an overall "crowding out" phenomenon of the Chinese property market bubble on corporate R&D investment.

## Conclusions and implications

This study comprehensively employs the bubble measurement and structural vector autoregressive (SVAR) model to conduct empirical research on the dynamic transmission mechanism among the real estate bubble, financing constraints, and corporate innovation in China. The following two basic conclusions have been reached: (1) from 1998 to 2015 (except years 1999, 2009, and 2013), although Chinese property market was operating at a relative safety interval, its bubble level showed a trend of continuous expansion. Without major policy adjustments, in the next few years, the real estate bubble in China will be at risk of further increase and deviation from the safety interval. (2) The results of the impulse response and variance decomposition analysis based on the SVAR model indicate that the corporate R&D investment not only explains most of its impact response but also suffers from the long-term negative impact of the real estate bubble. The long-term "crowding-out effect" from the real estate bubble is far greater than the short-term "crowding-in effect." In other words, the continuously expanding real estate bubble will weaken the R&D intensity of enterprises for a long time, thereby further deepening the “low-tech lock-in” state of enterprises.

In the context of the rapid downward pressure on the economy in China, stable corporate R&D investment is particularly critical as an important segment of the entire economic transformation process. This study concludes that the booming real estate bubble will hinder the R&D investment of industrial enterprises and cause enterprises to fall into the so-called "low-tech lock-in" state for a long period, leaving the economic development in China to flounder. Therefore, the central and local governments must take the following regulatory measures: (1) promote the supply-side reform on the real estate market to head off the expansion of the property market bubble. The high inventory and bubble expansion in real estate, to a great extent, are caused by structural contradictions such as the "dislocation between supply and demand." We believe that it is not enough to rely only on stimulating consumption to resolve inventories and bubbles. More efforts are required to accelerate a series of supply-side structural reforms to bail out this economic plight. Measures, such as expanding government purchases and digestion, optimizing the structure of residential development, and encouraging mergers and reorganization of housing enterprises, should be proposed to address the imbalances in the supply structure of the real estate market and thus promote the healthy and stable development of the property market. (2) Loosen the entry conditions of private capital and bridge the profit gap among industries. For industries with excess profits (the real estate industry), the entry conditions for private capital should be further loosened to accelerate profit equalization among industries, thereby contributing to competition and innovation. (3) Promote financial system reform and improve the financing efficiency of enterprises. The financing constraints of enterprises caused by the information asymmetry have restrained the innovation input of enterprises to a certain extent, which is one of the reasons that induce the cross-industry arbitrage of enterprises. Therefore, it becomes imperative to promote the financial system reform and improve the financing efficiency of enterprises. Due to data acquisition limitations, the evaluation index systems of PM have usually been selected and then categorised into three groups: supply side risk, demand side risk, and financial risk. However, it does not cover more direct factors attributed to the “real estate bubble”. Different indicator selections may cause deviations of the results. Moreover, the combination evaluation model is essentially data driven, not based on economic theoretical foundation. In the future, more data will be collected and new indicators and methods will be investigated to enhance the study depth.

## Supporting information

S1 Data (XLS)
